# Crystal structure of [(1,2,3,4,11,12-η)-anthracene]tris­(tri­methyl­stann­yl)cobalt(III)

**DOI:** 10.1107/S1600536814021709

**Published:** 2014-10-08

**Authors:** William W. Brennessel, John E. Ellis

**Affiliations:** a Department of Chemistry, 120 Trustee Road, University of Rochester, Rochester, NY 14627, USA; b Department of Chemistry, 207 Pleasant Street SE, University of Minnesota, Minneapolis, MN 55455, USA

**Keywords:** crystal structure, cobalt, anthracene, tri­methyl­stannyl ligands, flat-slipped coordination mode, NMR data

## Abstract

The asymmetric unit of the title structure, [Co(η^6^-C_14_H_10_){Sn(CH_3_)_3_}_3_], contains two independent mol­ecules. Each anthracene ligand is η^6^-coordinating to a Co^III^ cation and is nearly planar [fold angles of 5.4 (3) and 9.7 (3)°], as would be expected for its behaving almost entirely as a donor to a high-oxidation-state metal center. The slight fold in each anthracene ligand gives rise to slightly longer Co—C bond lengths to the ring junction carbon atoms than to the other four. Each Co^III^ cation is further coordinated by three Sn(CH_3_)_3_ ligands, giving each mol­ecule a three-legged piano-stool geometry. In each of the two independent mol­ecules, the trio of SnMe_3_ ligands are modeled as disordered over two positions, rotated by approximately 30%, such that the C atoms nearly overlap. In one mol­ecule, the disorder ratio refined to 0.9365 (8):0.0635 (8), while that for the other refined to 0.9686 (8):0.0314 (8). The mol­ecules are well separated, and thus no significant inter­molecular inter­actions are observed. The compound is of inter­est as the first structure report of an η^6^-anthracene cobalt(III) complex.

## Abstract 2

The first reported structure of a cobalt complex containing an η^6^-anthracene ligand is presented. The anthracene ligand is nearly flat and coordinates the metal asymmetrically, such that the ring junction carbon atoms are slightly further from the cobalt center than are the other four.

## Chemical context   

Oxidation derivatives of unstable low-valent species often provide indirect support for their formulations. For example, thermally unstable alkyl isocyanide complexes of formally *M*(−II) that were proposed to be ‘K_2_[*M*(CN*t*Bu)_4_],’ *M* = Fe (Brennessel *et al.*, 2007 ▶), Ru (Corella *et al.*, 1992 ▶), were reacted at low temperature *in situ* with SnPh_3_Cl to afford isolable and readily characterizable derivatives, *trans*-*M*(SnPh_3_)_2_(CN*t*Bu)_4_. Similarly, it was planned to derivatize the formally Co(−I) anion [Co(C_10_H_8_)_2_]^−^, C_10_H_8_ = naphthalene, which is the analog of the well-characterized and isolable anthracene cobaltate [Co(C_14_H_10_)_2_]^−^ (C_14_H_10_ = anthracene; Brennessel *et al.*, 2002 ▶). To date, the only established instance of [Co(C_10_H_8_)_2_]^−^ is as part of the highly specific triple salt [K(18-crown-6)]_3_[Co(C_10_H_8_)(C_2_H_4_)_2_]_2_[Co(C_10_H_8_)_2_] (Brennes­sel *et al.*, 2006 ▶). But before applying this procedure to the naphthalene system, we chose to first apply it to the well-behaved anthracene system to test the feasibility of the deriv­atization. Thus, one equivalent of SnMe_3_Cl was added *in situ* to a THF solution of [K(THF)_*x*_][Co(C_14_H_10_)_2_] (Brennessel *et al.*, 2002 ▶), which produced an intense violet, pentane-soluble species. Rather than being the expected ‘[Co(C_14_H_10_)_2_(SnMe_3_)]’ formally Co(I) species, however, after further investigation it was determined to be the title compound, [Co(η^6^-C_14_H_10_)(SnMe_3_)_3_] (I), based on single-crystal X-ray diffraction.
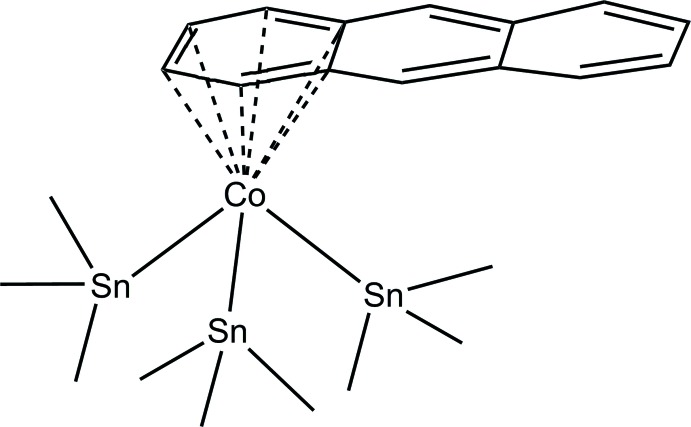


Similar reactions using SnPh_3_ and Sn(cyclo­hex­yl)_3_ produced only intra­ctable mixtures. Filtration of the reaction mixture left a very reactive dark-gray filter cake, which appeared to be from the deposition of Co metal. A tentative balanced equation has been proposed based on the initial evidence (see equation below). No yield was obtained, but if the equation holds, a qu­anti­tative yield would only be 33.3% based on cobalt. Single crystals were grown from a saturated pentane solution in a 243 K freezer and NMR experiments (see *Synthesis and crystallization*) were performed on the single crystals, which corroborated the structure analysis from diffraction data.
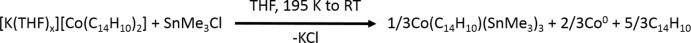


## Structural Commentary   

**Figure 1:**
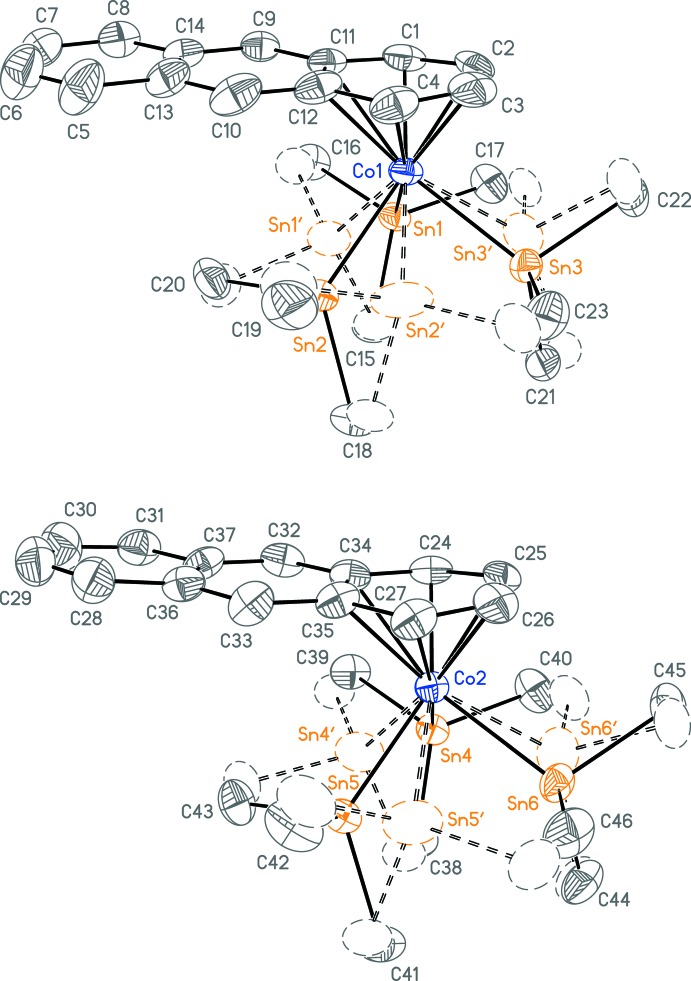
The two independent mol­ecules of (I), showing the atom numbering. The minor components of the disorder are shown with dashed lines and boundary ellipsoids. The two orientations of the SnMe_3_ ligand set fit in essentially the same volume because the methyl groups are overlapped. Displacement ellipsoids are drawn at the 50% probability level and hydrogen atoms have been omitted.

The structure contains two independent mol­ecules of (I) (Fig. 1 ▶) that are metrically very similar. Each mol­ecule contains one anthracene and three SnMe_3_ ligands in a three-legged-piano-stool geometry. In each of the two independent mol­ecules, the trio of tin ligands are disordered with a 30° rotation of the set, although the minor component of the disorder is very small (<10% by mass in both cases). The anthracene ligands in both mol­ecules are coordinated in an η^6^ mode and are nearly planar, with only the slightest bends at the imaginary lines joining atoms C1 and C4 [5.4 (3)°] and C24 and C27 [9.7 (3)°]. The Co—C distances to the ring junction carbon atoms are slightly longer by 0.17 Å than those to the metal-coordinating non-ring junction atoms (Table 1 ▶). This has been referred to as a ‘flat-slipped’ coordination mode, and is likely due to an anti­bonding component of the anthracene HOMO at the ring-junction carbon atoms (Zhu *et al.*, 2006 ▶). Thus the anthracene ligand is displaced slightly from the symmetric coordination mode found in η^6^-benzene metal complexes, in order to maximize the bonding overlaps with the four non-ring-junction carbon atoms. Because the metal is formally *d* ^6^ Co^III^, the π-donation from the anthracene ligand is likely the most important contribution to its bonding.

## Database Survey   

Structures of η^6^-coordinated anthracene transition metal complexes are few [Cambridge Structural Database, Version 5.35, update No. 3, May 2014; Groom & Allen, 2014 ▶], but range from Ti (Seaburg *et al.*, 1998 ▶) to Co (this work). Although one ligand in the titanium complex, [Ti(dmpe)(η^4^-C_14_H_10_)(η^6^-C_14_H_10_)] [dmpe = 1,2-bis(dimethylphosphino), is considered η^6^-coordinating based on Ti—C bond lengths, the fold angle between the plane consisting of non-ring-junction metal-coordinating carbon atoms and the rest of the ligand is nearly 20°, very likely placing it on the cusp of an η^4^ coordination mode. However, both [Cr(C_14_H_10_)(CO)_3_] (Hanic & Mills, 1968 ▶) and [Mo(C_14_H_10_)(PMe_3_)_3_] (Zhu *et al.*, 2006 ▶) have nearly planar anthracene ligands (6.6 and 5.5–5.8°, respectively). The small fold angles and the *M*—C(ring junction) bond lengths that are slightly longer than the *M*—C(non-ring junction) ones exemplify the ‘flat-slipped’ coordination mode (Table 1 ▶). For these cases of early transition metals, the π-donation of anthracene is supplemented by δ-backbonding to the anthracene LUMO; however, the C—C bond lengths are not all that different from those seen in normal-valent late transition metal complexes, and all are elongated relative to those in free anthracene (Table 1 ▶).

In the structures of later transition metal compounds, the η^6^ ‘flat-slipped’ coordination mode is found in normal- or slightly sub-valent metal complexes, and the fold angle appears to be sensitive to oxidation state. In structures with Ru^II^ coordination centers (Garcia *et al.*, 2010 ▶; Konovalov *et al.*, 2011 ▶) the fold angles are 3.1 and 4.4°, respectively. As the oxidation state decreases, as in the cases of Fe^I^ (Schnöckelborg *et al.*, 2012 ▶; Hatanaka *et al.*, 2012 ▶) and Rh^I^ (Woolf *et al.*, 2011 ▶), the fold angles increase slightly to 15.8, 9.1, 9.2, and 13.8°, respectively. Although fold angles may be subject to a variety of additional effects, including packing and sterics, in general the trend is that these angles increase with greater electron-acceptor behavior. This has been examined for the series Cp*Fe(C_14_H_10_)(−/0/+) and Cp*Fe(C_10_H_8_)(−/0/+), Cp* = C_5_Me_5_, by a combination of X-ray crystallography and DFT methods (Schnöckelborg *et al.*, 2012 ▶). In low oxidation states, the fold angles are significant and the ring-junction carbon atoms are bent away from the metal, thus making the coordination η^4^. Whereas the folds become almost non-existent (<10°) for normal valent oxidation states and the coordination is η^6^, consistent with what is observed in (I), a formally Co^III^, *d* ^6^ metal atom.

The ^1^H NMR data trends are in agreement with those reported for the isoelectronic species, [RuCp(η^6^-C_14_H_10_)](PF_6_) (McNair & Mann, 1986 ▶) and [OsCp(η^6^-C_14_H_10_)](PF_6_) (Freedman *et al.*, 1997 ▶), and for the cationic cobalt complex [(η^4^-C_4_Me_4_)Co(η^6^-C_14_H_10_)](PF_6_) (Mutseneck *et al.*, 2007 ▶). The most upfield anthracene ^1^H NMR resonances of δ = 5.98 (I), 6.33 (Ru), 6.62 (Os), and 6.65 (Co cation) p.p.m. demonstrate that the ligand is behaving almost entirely as a donor. The slightly upfield shifts from those of free anthracene may be due to a synergistic effect caused by the donation from the other ligands present, especially three SnMe_3_^−^ anions, for which the shift is most pronounced.

To date, the analogous reaction using naphthalene instead of anthracene has not been performed.

## Synthesis and crystallization   

A clear blue solution of CoBr_2_ (0.500 g, 2.29 mmol) in THF (60 ml, 195 K) was added to a deep-blue solution of K[C_14_H_10_] (6.86 mmol) in THF (60 ml, 195 K). To the resulting deep pinkish-red solution was added SnMe_3_Cl (0.455 g, 2.29 mmol) in THF (20 ml, 195 K), which dulled the color. After slow warming to room temperature, the solution was filtered to remove KBr and KCl. The solvent was removed under vacuum, and the product was extracted into pentane (25 ml) and filtered to give an intense violet solution. After the filtrate was cooled to and kept at 273 K for one h, the violet supernatant was carefully transferred to another vessel and placed in a freezer (243 K) for two days, during which time big purple–black crystals of the title complex formed. No attempts to establish the yield or obtain bulk elemental analyses were carried out. However, the product was characterized using the single crystals in solution by NMR and in the solid state by single-crystal X-ray diffraction. ^1^H NMR (300 MHz, CDCl_3_, 293 K, δ, p.p.m.): 8.32 (*s*, 2H, H_9_,_10_), 7.90 (*m*, 2H, H_5_,_8_ or H_6_,_7_), 7.48 (*m*, 2H, H_5_,_8_ or H_6_,_7_), 7.27 (CDCl_3_), 6.79 (*m*, 2H, H_1_,_4_ or H_2_,_3_), 5.98 (*m*, 2H, H_1_,_4_ or H_2_,_3_), 0.01 [*s*, 27H, ^2^ *J*(^1^H^119^Sn) = 20.6 Hz, C*H*_3_], ^13^C{^1^H} NMR (75.5 MHz, CDCl_3_, 293 K, δ, p.p.m.): 127.8 (An), 127.4 (An), 126.9 (An), 93.3 (An), 86.3 (An), 77.2 (*t*, CDCl_3_), −2.9 (*C*H_3_). Quaternary carbon resonances were not resolved.

## Refinement   

Crystal data, data collection and structure refinement details are summarized in Table 2 ▶. In each of the two independent mol­ecules, the trio of SnMe_3_ ligands are modeled as disordered over two positions, such that the carbon atoms nearly overlap. In the mol­ecule containing Co1 the disorder ratio refined to 0.9366 (8):0.0634 (8). That for the other mol­ecule refined to 0.9685 (8):0.0315 (8). Despite the small fraction of the minor components, when the disorders are not modeled, the *R*1 residual increases from 0.0375 to 0.0538. For each disorder model, analogous bond lengths and angles were heavily restrained to be similar. Anisotropic displacement parameters for pairs of near-isopositional carbon atoms were constrained to be equivalent.

The rather large residual peak in the difference map (1.93 electrons per Å^3^, located 1.74 Å from atom C4) has no chemical meaning. It (and other similar smaller peaks) is likely due to a very minor twin component whose twin law is [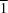 0 0 / 0 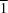 0 / −0.623 −0.754 1], a 180 degree rotation about [001] (Parsons *et al.*, 2003 ▶).

H-atom positions of cobalt-coordinating carbon atoms were refined freely, but with relative displacement parameters. All other H atoms were placed geometrically and treated as riding atoms: *sp* ^2^, C—H = 0.95 Å, with *U*_iso_(H) = 1.2*U*_eq_(C), and methyl, C—H = 0.98 Å with *U*_iso_(H) = 1.5*U*_eq_(C).

## Supplementary Material

Crystal structure: contains datablock(s) I, global. DOI: 10.1107/S1600536814021709/bg2536sup1.cif

Structure factors: contains datablock(s) I. DOI: 10.1107/S1600536814021709/bg2536Isup2.hkl

CCDC reference: 1027247

Additional supporting information:  crystallographic information; 3D view; checkCIF report

## Figures and Tables

**Figure 2:**
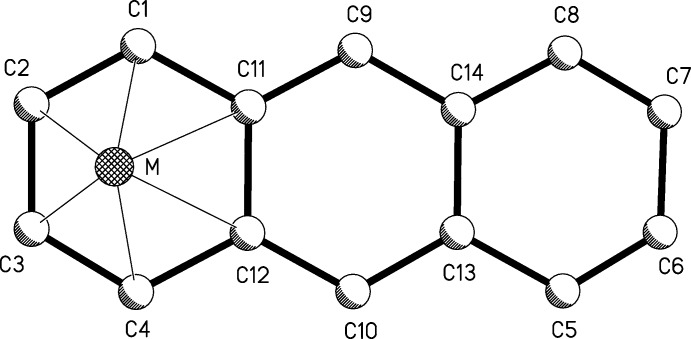
Anthracene numbering scheme for comparisons in Table 1 ▶.

**Table 1:** Comparison of (I) with free anthracene and selected ‘flat-slipped’ structures (, ) The numbering is according to Fig.2 ▶. For (I) and the molybdenum complex, only one of the two independent molecules for each is listed because they are metrically similar.

Feature	(I)	Anthracene*^*a*^*	[(Cp")Ru(An)][PF_6_]*^*b*^*	MoAn(PMe_3_)_3_ *^*c*^*
*M*C1	2.101(5)		2.207(4)	2.297(3)
*M*C2	2.102(5)		2.217(4)	2.261(3)
*M*C3	2.098(5)		2.223(4)	2.285(3)
*M*C4	2.132(5)		2.210(4)	2.268(3)
*M*C11	2.273(5)		2.289(4)	2.405(3)
*M*C21	2.274(5)		2.283(4)	2.424(3)
				
Increase (avg.)	0.165		0.072	0.137
				
C1C2	1.387(7)	1.3675(9)	1.399(6)	1.407(6)
C2C3	1.423(8)	1.4264(10)	1.415(7)	1.419(7)
C3C4	1.393(9)	1.3674(9)	1.398(7)	1.408(7)
C1C11	1.438(7)	1.4297(8)	1.431(6)	1.434(6)
C4C12	1.436(7)	1.4295(8)	1.441(6)	1.452(6)
C11C12	1.449(7)	1.4384(8)	1.449(5)	1.455(6)
				
Fold angle	5.4(3)		4.4	5.4

Notes: (*a*) unpublished structure determined locally; (*b*) Konovalov *et al.* (2011 ▶), Cp = C_5_Me_4_(CH_2_OMe), An = anthracene; (*c*) Zhu *et al.* (2006 ▶).

**Table 2:** Experimental details

Crystal data	
Chemical formula	[CoSn_3_(CH_3_)_9_(C_14_H_10_)]
*M*_r_	728.52
Crystal system, space group	Triclinic, *P* 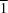
Temperature (K)	173
*a*, *b*, *c* ()	12.9784(18), 13.0834(18), 16.734(2)
, , ()	72.754(2), 75.891(2), 89.551(2)
*V* (^3^)	2625.5(6)
*Z*	4
Radiation type	Mo *K*
(mm^1^)	3.45
Crystal size (mm)	0.28 0.24 0.06

Data collection	
Diffractometer	Siemens SMART CCD platform
Absorption correction	Multi-scan (*SADABS*; Sheldrick, 2012 ▶)
*T*_min_, *T*_max_	0.493, 0.746
No. of measured, independent and observed [*I* > 2(*I*)] reflections	30588, 11891, 9564
*R*_int_	0.032
(sin /)_max_ (^1^)	0.650

Refinement	
*R*[*F* ^2^ > 2(*F* ^2^)], *wR*(*F* ^2^), *S*	0.038, 0.086, 1.08
No. of reflections	11891
No. of parameters	639
No. of restraints	42
H-atom treatment	H atoms treated by a mixture of independent and constrained refinement
_max_, _min_ (e ^3^)	1.93, 0.79

Computer programs: *SMART* and *SAINT* (Bruker, 2003 ▶), *SHELXS97*, *SHELXL2014* and *SHELXTL* (Sheldrick, 2008 ▶).

## supplementary crystallographic information

### Crystal data

**Table:**

[CoSn_3_(C_14_H_10_)(CH_3_)_9_]	*Z* = 4
*M**_r_* = 728.52	*F*(000) = 1408
Triclinic, *P*1	*D*_x_ = 1.843 Mg m^−^^3^
*a* = 12.9784 (18) Å	Mo *K*α radiation, λ = 0.71073 Å
*b* = 13.0834 (18) Å	Cell parameters from 3885 reflections
*c* = 16.734 (2) Å	µ = 3.45 mm^−^^1^
α = 72.754 (2)°	*T* = 173 K
β = 75.891 (2)°	Plate, dark purple
γ = 89.551 (2)°	0.28 × 0.24 × 0.06 mm
*V* = 2625.5 (6) Å^3^	

### Data collection

**Table:**

Siemens SMART CCD platform diffractometer	9564 reflections with *I* > 2σ(*I*)
Radiation source: normal-focus sealed tube	*R*_int_ = 0.032
ω scans	θ_max_ = 27.5°, θ_min_ = 1.3°
Absorption correction: multi-scan (*SADABS*; Sheldrick, 2012)	*h* = −16→16
*T*_min_ = 0.493, *T*_max_ = 0.746	*k* = −16→16
30588 measured reflections	*l* = −21→21
11891 independent reflections	

### Refinement

**Table:**

Refinement on *F*^2^	Primary atom site location: structure-invariant direct methods
Least-squares matrix: full	Secondary atom site location: difference Fourier map
*R*[*F*^2^ > 2σ(*F*^2^)] = 0.038	Hydrogen site location: mixed
*wR*(*F*^2^) = 0.086	H atoms treated by a mixture of independent and constrained refinement
*S* = 1.08	*w* = 1/[σ^2^(*F*_o_^2^) + (0.032*P*)^2^ + 4.7781*P*] where *P* = (*F*_o_^2^ + 2*F*_c_^2^)/3
11891 reflections	(Δ/σ)_max_ = 0.001
639 parameters	Δρ_max_ = 1.93 e Å^−^^3^
42 restraints	Δρ_min_ = −0.79 e Å^−^^3^

### Special details

**Table:**

Geometry. All e.s.d.'s (except the e.s.d. in the dihedral angle between two l.s. planes) are estimated using the full covariance matrix. The cell e.s.d.'s are taken into account individually in the estimation of e.s.d.'s in distances, angles and torsion angles; correlations between e.s.d.'s in cell parameters are only used when they are defined by crystal symmetry. An approximate (isotropic) treatment of cell e.s.d.'s is used for estimating e.s.d.'s involving l.s. planes.
Refinement. The largest residual peak of 1.93 electrons per Å^3^, located 1.74 Å from atom C4, has no chemical meaning. It (and other smaller peaks that likewise having no chemical meaning) is likely due to a very minor twin component whose twin law is [-1 0 0 / 0 - 1 0 / -0.623 - 0.754 1], a 180 degree rotation about [001] (Parsons, 2003).In each of the two independent molecules, the trio of SnMe_3_ ligands are modeled as disordered over two positions, such that the carbon atoms nearly overlap. In the molecule containing Co1 the disorder ratio refined to 0.9366 (8):0.0634 (8). That for the other molecule refined to 0.9686 (8):0.0314 (8). Despite the small mass of the minor components, when the disorders are not modeled, the R1 residual increases from 0.0375 to 0.0538.For each disorder model, analogous bond lengths and angles were heavily restrained to be similar. Anisotropic displacement parameters for pairs of near-isopositional carbon atoms were constrained to be equivalent.H atom positions of cobalt-coordinated carbon atoms were refined freely, but with relative thermal parameters as described below. All other H atoms were placed geometrically and treated as riding atoms: *sp*^2^, C—H = 0.95 Å, with *U*_iso_(H) = 1.2*U*_eq_(C), and methyl, C—H = 0.98 Å with *U*_iso_(H) = 1.5*U*_eq_(C).

### Fractional atomic coordinates and isotropic or equivalent isotropic displacement parameters (Å^2^)

**Table:**

	*x*	*y*	*z*	*U*_iso_*/*U*_eq_	Occ. (<1)
Co1	0.37703 (4)	0.13516 (5)	0.21652 (4)	0.02862 (14)	
C1	0.3223 (4)	0.0682 (4)	0.1335 (4)	0.0415 (12)	
H1	0.362 (4)	0.067 (4)	0.077 (4)	0.050*	
C2	0.2640 (4)	0.1551 (5)	0.1430 (4)	0.0461 (13)	
H2	0.260 (5)	0.209 (5)	0.097 (4)	0.055*	
C3	0.2146 (4)	0.1582 (5)	0.2279 (4)	0.0517 (15)	
H3	0.184 (5)	0.217 (5)	0.238 (4)	0.062*	
C4	0.2238 (4)	0.0760 (5)	0.3005 (4)	0.0450 (13)	
H4	0.196 (4)	0.083 (4)	0.359 (4)	0.054*	
C5	0.3347 (6)	−0.2885 (5)	0.4240 (4)	0.0660 (18)	
H5	0.3025	−0.2871	0.4811	0.079*	
C6	0.3857 (6)	−0.3749 (5)	0.4118 (5)	0.0712 (19)	
H6	0.3891	−0.4328	0.4611	0.085*	
C7	0.4344 (4)	−0.3834 (4)	0.3286 (4)	0.0463 (13)	
H7	0.4693	−0.4455	0.3219	0.056*	
C8	0.4293 (4)	−0.2988 (4)	0.2581 (4)	0.0458 (13)	
H8	0.4611	−0.3031	0.2018	0.055*	
C9	0.3733 (4)	−0.1173 (4)	0.1964 (3)	0.0354 (10)	
H9	0.4048	−0.1210	0.1398	0.042*	
C10	0.2779 (4)	−0.1078 (5)	0.3626 (4)	0.0506 (14)	
H10	0.2451	−0.1056	0.4194	0.061*	
C11	0.3238 (3)	−0.0242 (4)	0.2056 (3)	0.0351 (11)	
C12	0.2743 (4)	−0.0195 (4)	0.2919 (4)	0.0418 (12)	
C13	0.3286 (4)	−0.1992 (4)	0.3520 (3)	0.0453 (13)	
C14	0.3777 (4)	−0.2042 (4)	0.2670 (3)	0.0383 (11)	
Sn1	0.55882 (2)	0.16207 (3)	0.10994 (2)	0.03084 (9)	0.9365 (8)
C15	0.7078 (6)	0.2100 (7)	0.1306 (7)	0.0558 (16)	0.9365 (8)
H15A	0.7129	0.1689	0.1887	0.084*	0.9365 (8)
H15B	0.7100	0.2868	0.1249	0.084*	0.9365 (8)
H15C	0.7676	0.1960	0.0875	0.084*	0.9365 (8)
C16	0.6052 (8)	0.0186 (5)	0.0754 (4)	0.0454 (15)	0.9365 (8)
H16A	0.6783	0.0305	0.0390	0.068*	0.9365 (8)
H16B	0.5571	0.0015	0.0435	0.068*	0.9365 (8)
H16C	0.6011	−0.0412	0.1280	0.068*	0.9365 (8)
C17	0.5498 (5)	0.2734 (5)	−0.0128 (4)	0.0489 (14)	0.9365 (8)
H17A	0.6121	0.2687	−0.0581	0.073*	0.9365 (8)
H17B	0.5483	0.3466	−0.0086	0.073*	0.9365 (8)
H17C	0.4848	0.2553	−0.0272	0.073*	0.9365 (8)
Sn2	0.47432 (3)	0.10385 (3)	0.33620 (2)	0.03731 (10)	0.9365 (8)
C18	0.5817 (5)	0.2171 (6)	0.3523 (6)	0.0574 (18)	0.9365 (8)
H18A	0.6102	0.1824	0.4021	0.086*	0.9365 (8)
H18B	0.5432	0.2788	0.3620	0.086*	0.9365 (8)
H18C	0.6405	0.2414	0.3001	0.086*	0.9365 (8)
C19	0.3608 (8)	0.0671 (7)	0.4623 (4)	0.068 (2)	0.9365 (8)
H19A	0.3480	−0.0109	0.4879	0.102*	0.9365 (8)
H19B	0.2936	0.0987	0.4555	0.102*	0.9365 (8)
H19C	0.3897	0.0968	0.5003	0.102*	0.9365 (8)
C20	0.5637 (5)	−0.0349 (5)	0.3326 (5)	0.0568 (18)	0.9365 (8)
H20A	0.6340	−0.0231	0.3414	0.085*	0.9365 (8)
H20B	0.5721	−0.0467	0.2762	0.085*	0.9365 (8)
H20C	0.5256	−0.0980	0.3784	0.085*	0.9365 (8)
Sn3	0.39786 (3)	0.33197 (3)	0.20495 (2)	0.03421 (9)	0.9365 (8)
C21	0.5464 (5)	0.4278 (4)	0.1656 (4)	0.0454 (15)	0.9365 (8)
H21A	0.5320	0.5026	0.1609	0.068*	0.9365 (8)
H21B	0.5872	0.4232	0.1093	0.068*	0.9365 (8)
H21C	0.5876	0.4010	0.2085	0.068*	0.9365 (8)
C22	0.3205 (5)	0.4202 (5)	0.1061 (5)	0.0527 (17)	0.9365 (8)
H22A	0.2460	0.3927	0.1217	0.079*	0.9365 (8)
H22B	0.3571	0.4110	0.0503	0.079*	0.9365 (8)
H22C	0.3237	0.4965	0.1018	0.079*	0.9365 (8)
C23	0.3121 (5)	0.3570 (5)	0.3242 (4)	0.0522 (16)	0.9365 (8)
H23A	0.3552	0.3374	0.3664	0.078*	0.9365 (8)
H23B	0.2450	0.3123	0.3469	0.078*	0.9365 (8)
H23C	0.2970	0.4327	0.3137	0.078*	0.9365 (8)
Sn1'	0.5647 (4)	0.0882 (5)	0.1893 (4)	0.0500 (18)	0.0635 (8)
C15'	0.697 (7)	0.210 (8)	0.135 (7)	0.0558 (16)	0.0635 (8)
H15D	0.6906	0.2579	0.1709	0.084*	0.0635 (8)
H15E	0.7642	0.1749	0.1342	0.084*	0.0635 (8)
H15F	0.6956	0.2510	0.0762	0.084*	0.0635 (8)
C16'	0.604 (12)	−0.001 (6)	0.097 (4)	0.0454 (15)	0.0635 (8)
H16D	0.5901	0.0418	0.0419	0.068*	0.0635 (8)
H16E	0.6793	−0.0154	0.0875	0.068*	0.0635 (8)
H16F	0.5599	−0.0685	0.1191	0.068*	0.0635 (8)
C17'	0.601 (7)	−0.021 (6)	0.303 (3)	0.0522 (16)	0.0635 (8)
H17D	0.5864	0.0118	0.3499	0.078*	0.0635 (8)
H17E	0.5575	−0.0882	0.3209	0.078*	0.0635 (8)
H17F	0.6769	−0.0351	0.2893	0.078*	0.0635 (8)
Sn2'	0.3993 (5)	0.1957 (6)	0.3414 (4)	0.065 (2)	0.0635 (8)
C18'	0.554 (4)	0.231 (8)	0.356 (9)	0.0574 (18)	0.0635 (8)
H18D	0.6012	0.1747	0.3463	0.086*	0.0635 (8)
H18E	0.5850	0.3003	0.3139	0.086*	0.0635 (8)
H18F	0.5477	0.2353	0.4147	0.086*	0.0635 (8)
C19'	0.349 (9)	0.056 (5)	0.455 (4)	0.068 (2)	0.0635 (8)
H19D	0.3899	−0.0046	0.4461	0.102*	0.0635 (8)
H19E	0.3607	0.0724	0.5058	0.102*	0.0635 (8)
H19F	0.2728	0.0370	0.4649	0.102*	0.0635 (8)
C20'	0.307 (5)	0.326 (4)	0.364 (5)	0.0489 (14)	0.0635 (8)
H20D	0.2345	0.3151	0.3587	0.073*	0.0635 (8)
H20E	0.3029	0.3290	0.4229	0.073*	0.0635 (8)
H20F	0.3402	0.3939	0.3221	0.073*	0.0635 (8)
Sn3'	0.4487 (5)	0.3255 (5)	0.1205 (4)	0.0535 (18)	0.0635 (8)
C21'	0.542 (6)	0.450 (5)	0.137 (5)	0.0454 (15)	0.0635 (8)
H21D	0.5146	0.4561	0.1952	0.068*	0.0635 (8)
H21E	0.6165	0.4320	0.1290	0.068*	0.0635 (8)
H21F	0.5373	0.5184	0.0939	0.068*	0.0635 (8)
C22'	0.299 (4)	0.400 (6)	0.119 (6)	0.0527 (17)	0.0635 (8)
H22D	0.2647	0.4046	0.1773	0.079*	0.0635 (8)
H22E	0.3120	0.4729	0.0782	0.079*	0.0635 (8)
H22F	0.2515	0.3576	0.1021	0.079*	0.0635 (8)
C23'	0.516 (6)	0.320 (6)	−0.009 (2)	0.0568 (18)	0.0635 (8)
H23D	0.4769	0.2646	−0.0201	0.085*	0.0635 (8)
H23E	0.5120	0.3899	−0.0503	0.085*	0.0635 (8)
H23F	0.5912	0.3035	−0.0152	0.085*	0.0635 (8)
Co2	0.00682 (4)	0.36883 (5)	0.78274 (4)	0.02670 (13)	
C24	−0.0921 (4)	0.4357 (4)	0.8698 (3)	0.0363 (11)	
H24	−0.090 (4)	0.437 (4)	0.922 (3)	0.044*	
C25	−0.1491 (4)	0.3499 (4)	0.8624 (4)	0.0395 (11)	
H25	−0.181 (4)	0.292 (4)	0.914 (4)	0.047*	
C26	−0.1519 (4)	0.3471 (5)	0.7790 (4)	0.0429 (12)	
H26	−0.192 (4)	0.291 (4)	0.778 (3)	0.051*	
C27	−0.1030 (4)	0.4293 (4)	0.7063 (3)	0.0384 (11)	
H27	−0.103 (4)	0.422 (4)	0.652 (4)	0.046*	
C28	0.0525 (5)	0.8050 (5)	0.5773 (4)	0.0524 (14)	
H28	0.0434	0.8071	0.5222	0.063*	
C29	0.0933 (5)	0.8922 (5)	0.5886 (4)	0.0621 (17)	
H29	0.1127	0.9557	0.5408	0.074*	
C30	0.1081 (5)	0.8923 (5)	0.6696 (4)	0.0637 (17)	
H30	0.1393	0.9547	0.6748	0.076*	
C31	0.0788 (4)	0.8054 (4)	0.7391 (4)	0.0461 (13)	
H31	0.0888	0.8070	0.7931	0.055*	
C32	−0.0046 (4)	0.6209 (4)	0.8032 (3)	0.0381 (11)	
H32	0.0016	0.6222	0.8583	0.046*	
C33	−0.0198 (4)	0.6166 (4)	0.6414 (3)	0.0436 (12)	
H33	−0.0243	0.6151	0.5859	0.052*	
C34	−0.0510 (4)	0.5279 (4)	0.7965 (3)	0.0347 (10)	
C35	−0.0569 (4)	0.5249 (4)	0.7129 (3)	0.0358 (11)	
C36	0.0227 (4)	0.7085 (4)	0.6489 (3)	0.0390 (11)	
C37	0.0323 (4)	0.7102 (4)	0.7325 (3)	0.0379 (11)	
Sn4	0.12611 (2)	0.33492 (3)	0.88667 (2)	0.02952 (8)	0.9686 (8)
C38	0.2862 (4)	0.2827 (5)	0.8632 (4)	0.0469 (14)	0.9686 (8)
H38A	0.3119	0.2706	0.9155	0.070*	0.9686 (8)
H38B	0.3331	0.3381	0.8154	0.070*	0.9686 (8)
H38C	0.2862	0.2159	0.8482	0.070*	0.9686 (8)
C39	0.1534 (4)	0.4762 (5)	0.9231 (4)	0.0424 (14)	0.9686 (8)
H39A	0.2024	0.4609	0.9608	0.064*	0.9686 (8)
H39B	0.0856	0.4957	0.9540	0.064*	0.9686 (8)
H39C	0.1846	0.5357	0.8711	0.064*	0.9686 (8)
C40	0.0413 (5)	0.2233 (5)	1.0099 (4)	0.0519 (15)	0.9686 (8)
H40A	0.0801	0.2214	1.0537	0.078*	0.9686 (8)
H40B	0.0357	0.1515	1.0038	0.078*	0.9686 (8)
H40C	−0.0303	0.2467	1.0278	0.078*	0.9686 (8)
Sn5	0.17472 (2)	0.40061 (3)	0.65953 (2)	0.03177 (9)	0.9686 (8)
C41	0.2885 (5)	0.2841 (5)	0.6367 (4)	0.0550 (17)	0.9686 (8)
H41A	0.3471	0.3187	0.5864	0.083*	0.9686 (8)
H41B	0.2532	0.2255	0.6259	0.083*	0.9686 (8)
H41C	0.3166	0.2555	0.6877	0.083*	0.9686 (8)
C42	0.1347 (5)	0.4509 (6)	0.5354 (4)	0.0554 (17)	0.9686 (8)
H42A	0.1888	0.4292	0.4926	0.083*	0.9686 (8)
H42B	0.1322	0.5291	0.5165	0.083*	0.9686 (8)
H42C	0.0650	0.4172	0.5413	0.083*	0.9686 (8)
C43	0.2710 (5)	0.5340 (5)	0.6633 (4)	0.0466 (14)	0.9686 (8)
H43A	0.3367	0.5473	0.6169	0.070*	0.9686 (8)
H43B	0.2887	0.5162	0.7193	0.070*	0.9686 (8)
H43C	0.2308	0.5984	0.6555	0.070*	0.9686 (8)
Sn6	0.01690 (3)	0.17235 (3)	0.79114 (2)	0.03626 (9)	0.9686 (8)
C44	0.1420 (5)	0.0721 (5)	0.8241 (5)	0.0539 (16)	0.9686 (8)
H44A	0.1251	−0.0008	0.8243	0.081*	0.9686 (8)
H44B	0.1483	0.0706	0.8816	0.081*	0.9686 (8)
H44C	0.2095	0.1010	0.7814	0.081*	0.9686 (8)
C45	−0.1180 (5)	0.0839 (5)	0.8929 (5)	0.0530 (16)	0.9686 (8)
H45A	−0.1193	0.0083	0.8948	0.080*	0.9686 (8)
H45B	−0.1841	0.1140	0.8813	0.080*	0.9686 (8)
H45C	−0.1116	0.0894	0.9487	0.080*	0.9686 (8)
C46	−0.0068 (5)	0.1551 (5)	0.6712 (4)	0.0544 (16)	0.9686 (8)
H46A	−0.0268	0.0799	0.6798	0.082*	0.9686 (8)
H46B	0.0594	0.1780	0.6255	0.082*	0.9686 (8)
H46C	−0.0636	0.1998	0.6544	0.082*	0.9686 (8)
Sn4'	0.1818 (9)	0.4147 (9)	0.8092 (8)	0.053 (4)	0.0314 (8)
C38'	0.295 (8)	0.293 (7)	0.830 (8)	0.0469 (14)	0.0314 (8)
H38D	0.2997	0.2535	0.7876	0.070*	0.0314 (8)
H38E	0.3649	0.3275	0.8218	0.070*	0.0314 (8)
H38F	0.2711	0.2437	0.8883	0.070*	0.0314 (8)
C39'	0.191 (11)	0.485 (8)	0.910 (5)	0.0424 (14)	0.0314 (8)
H39D	0.1423	0.5430	0.9086	0.064*	0.0314 (8)
H39E	0.1699	0.4299	0.9662	0.064*	0.0314 (8)
H39F	0.2637	0.5137	0.8997	0.064*	0.0314 (8)
C40'	0.264 (10)	0.540 (6)	0.693 (4)	0.0466 (14)	0.0314 (8)
H40D	0.2168	0.5980	0.6797	0.070*	0.0314 (8)
H40E	0.3284	0.5677	0.7021	0.070*	0.0314 (8)
H40F	0.2833	0.5099	0.6444	0.070*	0.0314 (8)
Sn5'	0.1087 (11)	0.3209 (11)	0.6522 (7)	0.061 (4)	0.0314 (8)
C41'	0.279 (2)	0.305 (11)	0.619 (11)	0.0550 (17)	0.0314 (8)
H41D	0.2974	0.2480	0.6654	0.083*	0.0314 (8)
H41E	0.2994	0.2872	0.5649	0.083*	0.0314 (8)
H41F	0.3163	0.3730	0.6124	0.083*	0.0314 (8)
C42'	0.096 (12)	0.450 (7)	0.539 (6)	0.0554 (17)	0.0314 (8)
H42D	0.0212	0.4672	0.5448	0.083*	0.0314 (8)
H42E	0.1387	0.5141	0.5346	0.083*	0.0314 (8)
H42F	0.1218	0.4283	0.4871	0.083*	0.0314 (8)
C43'	0.040 (11)	0.176 (6)	0.641 (9)	0.0544 (16)	0.0314 (8)
H43D	−0.0375	0.1765	0.6549	0.082*	0.0314 (8)
H43E	0.0667	0.1729	0.5816	0.082*	0.0314 (8)
H43F	0.0610	0.1128	0.6811	0.082*	0.0314 (8)
Sn6'	0.0243 (11)	0.1762 (9)	0.8696 (8)	0.067 (5)	0.0314 (8)
C44'	0.152 (7)	0.078 (9)	0.838 (9)	0.0539 (16)	0.0314 (8)
H44D	0.2201	0.1143	0.8320	0.081*	0.0314 (8)
H44E	0.1420	0.0089	0.8834	0.081*	0.0314 (8)
H44F	0.1517	0.0652	0.7827	0.081*	0.0314 (8)
C45'	−0.111 (6)	0.074 (9)	0.877 (9)	0.0530 (16)	0.0314 (8)
H45D	−0.1767	0.1088	0.8912	0.080*	0.0314 (8)
H45E	−0.1033	0.0613	0.8207	0.080*	0.0314 (8)
H45F	−0.1130	0.0051	0.9214	0.080*	0.0314 (8)
C46'	0.012 (11)	0.178 (11)	1.001 (3)	0.0519 (15)	0.0314 (8)
H46D	−0.0454	0.2222	1.0179	0.078*	0.0314 (8)
H46E	−0.0030	0.1045	1.0407	0.078*	0.0314 (8)
H46F	0.0796	0.2077	1.0044	0.078*	0.0314 (8)

### Atomic displacement parameters (Å^2^)

**Table:**

	*U*^11^	*U*^22^	*U*^33^	*U*^12^	*U*^13^	*U*^23^
Co1	0.0235 (3)	0.0335 (3)	0.0337 (3)	0.0020 (2)	−0.0095 (2)	−0.0155 (3)
C1	0.033 (3)	0.057 (3)	0.048 (3)	0.001 (2)	−0.018 (2)	−0.030 (3)
C2	0.033 (3)	0.054 (3)	0.068 (4)	0.009 (2)	−0.032 (3)	−0.026 (3)
C3	0.022 (2)	0.061 (4)	0.084 (5)	0.006 (2)	−0.014 (3)	−0.040 (4)
C4	0.031 (3)	0.050 (3)	0.054 (3)	−0.006 (2)	0.000 (2)	−0.025 (3)
C5	0.094 (5)	0.048 (4)	0.042 (3)	−0.011 (3)	0.000 (3)	−0.006 (3)
C6	0.090 (5)	0.048 (4)	0.061 (4)	−0.007 (3)	−0.009 (4)	−0.002 (3)
C7	0.044 (3)	0.045 (3)	0.048 (3)	−0.001 (2)	−0.004 (2)	−0.018 (3)
C8	0.040 (3)	0.050 (3)	0.051 (3)	0.003 (2)	−0.008 (2)	−0.023 (3)
C9	0.032 (2)	0.044 (3)	0.035 (3)	0.000 (2)	−0.0075 (19)	−0.019 (2)
C10	0.050 (3)	0.051 (3)	0.044 (3)	−0.017 (3)	0.008 (2)	−0.020 (3)
C11	0.026 (2)	0.041 (3)	0.045 (3)	−0.0019 (19)	−0.011 (2)	−0.021 (2)
C12	0.029 (2)	0.048 (3)	0.051 (3)	−0.007 (2)	0.001 (2)	−0.026 (3)
C13	0.045 (3)	0.043 (3)	0.042 (3)	−0.012 (2)	0.001 (2)	−0.012 (2)
C14	0.033 (2)	0.043 (3)	0.042 (3)	−0.006 (2)	−0.008 (2)	−0.018 (2)
Sn1	0.02348 (16)	0.03726 (19)	0.03407 (19)	0.00299 (13)	−0.00831 (13)	−0.01343 (15)
C15	0.030 (3)	0.064 (4)	0.082 (5)	0.002 (3)	−0.017 (3)	−0.034 (3)
C16	0.037 (3)	0.055 (4)	0.048 (4)	0.011 (3)	−0.006 (3)	−0.026 (3)
C17	0.041 (3)	0.058 (4)	0.040 (3)	0.004 (3)	−0.008 (2)	−0.006 (3)
Sn2	0.0451 (2)	0.0371 (2)	0.0365 (2)	0.00380 (15)	−0.02069 (16)	−0.01288 (16)
C18	0.064 (4)	0.062 (4)	0.063 (4)	0.003 (3)	−0.039 (4)	−0.027 (3)
C19	0.092 (5)	0.076 (5)	0.038 (3)	−0.007 (4)	−0.020 (3)	−0.015 (3)
C20	0.065 (5)	0.054 (4)	0.066 (4)	0.024 (3)	−0.039 (4)	−0.021 (3)
Sn3	0.03129 (18)	0.03110 (18)	0.0436 (2)	0.00540 (13)	−0.01187 (15)	−0.01455 (15)
C21	0.040 (3)	0.035 (3)	0.059 (4)	−0.004 (2)	−0.018 (3)	−0.005 (3)
C22	0.047 (3)	0.047 (4)	0.064 (4)	0.015 (3)	−0.020 (3)	−0.012 (3)
C23	0.055 (4)	0.050 (4)	0.055 (4)	0.013 (3)	−0.009 (3)	−0.026 (3)
Sn1'	0.037 (3)	0.061 (4)	0.059 (4)	0.003 (3)	−0.013 (3)	−0.027 (3)
C15'	0.030 (3)	0.064 (4)	0.082 (5)	0.002 (3)	−0.017 (3)	−0.034 (3)
C16'	0.037 (3)	0.055 (4)	0.048 (4)	0.011 (3)	−0.006 (3)	−0.026 (3)
C17'	0.055 (4)	0.050 (4)	0.055 (4)	0.013 (3)	−0.009 (3)	−0.026 (3)
Sn2'	0.066 (4)	0.084 (5)	0.051 (4)	−0.022 (4)	−0.011 (3)	−0.034 (4)
C18'	0.064 (4)	0.062 (4)	0.063 (4)	0.003 (3)	−0.039 (4)	−0.027 (3)
C19'	0.092 (5)	0.076 (5)	0.038 (3)	−0.007 (4)	−0.020 (3)	−0.015 (3)
C20'	0.041 (3)	0.058 (4)	0.040 (3)	0.004 (3)	−0.008 (2)	−0.006 (3)
Sn3'	0.046 (4)	0.060 (4)	0.054 (4)	0.008 (3)	−0.017 (3)	−0.012 (3)
C21'	0.040 (3)	0.035 (3)	0.059 (4)	−0.004 (2)	−0.018 (3)	−0.005 (3)
C22'	0.047 (3)	0.047 (4)	0.064 (4)	0.015 (3)	−0.020 (3)	−0.012 (3)
C23'	0.065 (5)	0.054 (4)	0.066 (4)	0.024 (3)	−0.039 (4)	−0.021 (3)
Co2	0.0242 (3)	0.0307 (3)	0.0301 (3)	0.0025 (2)	−0.0111 (2)	−0.0132 (3)
C24	0.032 (2)	0.047 (3)	0.036 (3)	0.015 (2)	−0.010 (2)	−0.021 (2)
C25	0.021 (2)	0.042 (3)	0.051 (3)	0.0059 (19)	−0.005 (2)	−0.010 (2)
C26	0.027 (2)	0.052 (3)	0.061 (4)	0.003 (2)	−0.024 (2)	−0.024 (3)
C27	0.040 (3)	0.044 (3)	0.044 (3)	0.011 (2)	−0.027 (2)	−0.019 (2)
C28	0.067 (4)	0.047 (3)	0.039 (3)	0.005 (3)	−0.004 (3)	−0.014 (3)
C29	0.073 (4)	0.047 (4)	0.051 (4)	−0.001 (3)	0.000 (3)	−0.006 (3)
C30	0.067 (4)	0.049 (4)	0.071 (4)	−0.011 (3)	−0.004 (3)	−0.023 (3)
C31	0.040 (3)	0.049 (3)	0.058 (3)	0.004 (2)	−0.012 (2)	−0.030 (3)
C32	0.034 (2)	0.056 (3)	0.035 (3)	0.007 (2)	−0.012 (2)	−0.026 (2)
C33	0.059 (3)	0.044 (3)	0.034 (3)	0.011 (2)	−0.020 (2)	−0.015 (2)
C34	0.029 (2)	0.045 (3)	0.034 (3)	0.009 (2)	−0.0116 (19)	−0.016 (2)
C35	0.037 (3)	0.040 (3)	0.041 (3)	0.011 (2)	−0.022 (2)	−0.020 (2)
C36	0.041 (3)	0.043 (3)	0.037 (3)	0.012 (2)	−0.011 (2)	−0.016 (2)
C37	0.036 (3)	0.037 (3)	0.051 (3)	0.013 (2)	−0.022 (2)	−0.021 (2)
Sn4	0.02549 (16)	0.03679 (18)	0.03028 (17)	0.00225 (12)	−0.01220 (12)	−0.01196 (14)
C38	0.033 (3)	0.059 (4)	0.059 (4)	0.011 (2)	−0.022 (3)	−0.024 (3)
C39	0.038 (3)	0.054 (3)	0.050 (3)	0.004 (2)	−0.020 (2)	−0.029 (3)
C40	0.048 (3)	0.064 (4)	0.036 (3)	−0.004 (3)	−0.014 (2)	0.000 (3)
Sn5	0.02953 (17)	0.03985 (19)	0.02961 (17)	0.00062 (13)	−0.00817 (13)	−0.01540 (14)
C41	0.049 (3)	0.059 (4)	0.059 (4)	0.014 (3)	−0.006 (3)	−0.027 (3)
C42	0.052 (4)	0.089 (5)	0.032 (3)	0.002 (3)	−0.017 (3)	−0.023 (3)
C43	0.043 (3)	0.051 (3)	0.045 (3)	−0.012 (2)	−0.013 (3)	−0.012 (3)
Sn6	0.03465 (18)	0.03089 (18)	0.0499 (2)	0.00055 (13)	−0.01780 (15)	−0.01638 (16)
C44	0.060 (4)	0.035 (3)	0.076 (5)	0.014 (3)	−0.031 (3)	−0.019 (3)
C45	0.042 (3)	0.046 (3)	0.070 (4)	−0.009 (2)	−0.021 (3)	−0.009 (3)
C46	0.060 (4)	0.055 (4)	0.066 (4)	0.004 (3)	−0.029 (3)	−0.036 (3)
Sn4'	0.038 (6)	0.074 (9)	0.062 (9)	0.009 (6)	−0.026 (6)	−0.032 (7)
C38'	0.033 (3)	0.059 (4)	0.059 (4)	0.011 (2)	−0.022 (3)	−0.024 (3)
C39'	0.038 (3)	0.054 (3)	0.050 (3)	0.004 (2)	−0.020 (2)	−0.029 (3)
C40'	0.043 (3)	0.051 (3)	0.045 (3)	−0.012 (2)	−0.013 (3)	−0.012 (3)
Sn5'	0.059 (8)	0.082 (10)	0.064 (9)	0.016 (7)	−0.026 (7)	−0.046 (8)
C41'	0.049 (3)	0.059 (4)	0.059 (4)	0.014 (3)	−0.006 (3)	−0.027 (3)
C42'	0.052 (4)	0.089 (5)	0.032 (3)	0.002 (3)	−0.017 (3)	−0.023 (3)
C43'	0.060 (4)	0.055 (4)	0.066 (4)	0.004 (3)	−0.029 (3)	−0.036 (3)
Sn6'	0.067 (9)	0.053 (9)	0.072 (10)	0.000 (7)	−0.021 (8)	−0.005 (7)
C44'	0.060 (4)	0.035 (3)	0.076 (5)	0.014 (3)	−0.031 (3)	−0.019 (3)
C45'	0.042 (3)	0.046 (3)	0.070 (4)	−0.009 (2)	−0.021 (3)	−0.009 (3)
C46'	0.048 (3)	0.064 (4)	0.036 (3)	−0.004 (3)	−0.014 (2)	0.000 (3)

### Geometric parameters (Å, º)

**Table:**

Co1—C3	2.098 (5)	Co2—C24	2.088 (5)
Co1—C1	2.101 (5)	Co2—C26	2.100 (5)
Co1—C2	2.102 (5)	Co2—C25	2.104 (5)
Co1—C4	2.132 (5)	Co2—C27	2.136 (5)
Co1—C11	2.273 (5)	Co2—C34	2.264 (5)
Co1—C12	2.274 (5)	Co2—C35	2.291 (5)
Co1—Sn1'	2.474 (5)	Co2—Sn5'	2.512 (11)
Co1—Sn2'	2.521 (5)	Co2—Sn4'	2.526 (10)
Co1—Sn3	2.5359 (8)	Co2—Sn6	2.5366 (8)
Co1—Sn1	2.5418 (7)	Co2—Sn4	2.5423 (7)
Co1—Sn2	2.5518 (7)	Co2—Sn5	2.5471 (7)
Co1—Sn3'	2.566 (6)	Co2—Sn6'	2.549 (12)
C1—C2	1.387 (7)	C24—C25	1.400 (7)
C1—C11	1.438 (7)	C24—C34	1.435 (7)
C1—H1	0.97 (5)	C24—H24	0.89 (5)
C2—C3	1.423 (8)	C25—C26	1.416 (8)
C2—H2	0.88 (6)	C25—H25	0.96 (5)
C3—C4	1.393 (9)	C26—C27	1.377 (8)
C3—H3	0.91 (6)	C26—H26	0.91 (6)
C4—C12	1.436 (7)	C27—C35	1.435 (7)
C4—H4	0.99 (6)	C27—H27	0.94 (5)
C5—C6	1.348 (9)	C28—C29	1.343 (8)
C5—C13	1.425 (8)	C28—C36	1.437 (7)
C5—H5	0.9500	C28—H28	0.9500
C6—C7	1.418 (8)	C29—C30	1.417 (9)
C6—H6	0.9500	C29—H29	0.9500
C7—C8	1.373 (8)	C30—C31	1.340 (8)
C7—H7	0.9500	C30—H30	0.9500
C8—C14	1.431 (7)	C31—C37	1.434 (7)
C8—H8	0.9500	C31—H31	0.9500
C9—C14	1.388 (7)	C32—C37	1.382 (7)
C9—C11	1.403 (7)	C32—C34	1.406 (7)
C9—H9	0.9500	C32—H32	0.9500
C10—C13	1.397 (8)	C33—C36	1.378 (7)
C10—C12	1.398 (8)	C33—C35	1.407 (7)
C10—H10	0.9500	C33—H33	0.9500
C11—C12	1.449 (7)	C34—C35	1.432 (6)
C13—C14	1.431 (7)	C36—C37	1.440 (7)
Sn1—C16	2.162 (5)	Sn4—C38	2.163 (5)
Sn1—C17	2.167 (6)	Sn4—C39	2.167 (5)
Sn1—C15	2.174 (6)	Sn4—C40	2.175 (5)
C15—H15A	0.9800	C38—H38A	0.9800
C15—H15B	0.9800	C38—H38B	0.9800
C15—H15C	0.9800	C38—H38C	0.9800
C16—H16A	0.9800	C39—H39A	0.9800
C16—H16B	0.9800	C39—H39B	0.9800
C16—H16C	0.9800	C39—H39C	0.9800
C17—H17A	0.9800	C40—H40A	0.9800
C17—H17B	0.9800	C40—H40B	0.9800
C17—H17C	0.9800	C40—H40C	0.9800
Sn2—C20	2.155 (6)	Sn5—C41	2.164 (6)
Sn2—C18	2.159 (6)	Sn5—C42	2.174 (5)
Sn2—C19	2.179 (7)	Sn5—C43	2.179 (5)
C18—H18A	0.9800	C41—H41A	0.9800
C18—H18B	0.9800	C41—H41B	0.9800
C18—H18C	0.9800	C41—H41C	0.9800
C19—H19A	0.9800	C42—H42A	0.9800
C19—H19B	0.9800	C42—H42B	0.9800
C19—H19C	0.9800	C42—H42C	0.9800
C20—H20A	0.9800	C43—H43A	0.9800
C20—H20B	0.9800	C43—H43B	0.9800
C20—H20C	0.9800	C43—H43C	0.9800
Sn3—C23	2.151 (6)	Sn6—C44	2.152 (6)
Sn3—C21	2.157 (5)	Sn6—C45	2.172 (6)
Sn3—C22	2.176 (6)	Sn6—C46	2.177 (6)
C21—H21A	0.9800	C44—H44A	0.9800
C21—H21B	0.9800	C44—H44B	0.9800
C21—H21C	0.9800	C44—H44C	0.9800
C22—H22A	0.9800	C45—H45A	0.9800
C22—H22B	0.9800	C45—H45B	0.9800
C22—H22C	0.9800	C45—H45C	0.9800
C23—H23A	0.9800	C46—H46A	0.9800
C23—H23B	0.9800	C46—H46B	0.9800
C23—H23C	0.9800	C46—H46C	0.9800
Sn1'—C16'	2.162 (7)	Sn4'—C38'	2.163 (6)
Sn1'—C17'	2.167 (7)	Sn4'—C39'	2.167 (6)
Sn1'—C15'	2.174 (7)	Sn4'—C40'	2.175 (7)
C15'—H15D	0.9800	C38'—H38D	0.9800
C15'—H15E	0.9800	C38'—H38E	0.9800
C15'—H15F	0.9800	C38'—H38F	0.9800
C16'—H16D	0.9800	C39'—H39D	0.9800
C16'—H16E	0.9800	C39'—H39E	0.9800
C16'—H16F	0.9800	C39'—H39F	0.9800
C17'—H17D	0.9800	C40'—H40D	0.9800
C17'—H17E	0.9800	C40'—H40E	0.9800
C17'—H17F	0.9800	C40'—H40F	0.9800
Sn2'—C20'	2.155 (7)	Sn5'—C41'	2.164 (7)
Sn2'—C18'	2.159 (7)	Sn5'—C42'	2.174 (7)
Sn2'—C19'	2.179 (8)	Sn5'—C43'	2.179 (7)
C18'—H18D	0.9800	C41'—H41D	0.9800
C18'—H18E	0.9800	C41'—H41E	0.9800
C18'—H18F	0.9800	C41'—H41F	0.9800
C19'—H19D	0.9800	C42'—H42D	0.9800
C19'—H19E	0.9800	C42'—H42E	0.9800
C19'—H19F	0.9800	C42'—H42F	0.9800
C20'—H20D	0.9800	C43'—H43D	0.9800
C20'—H20E	0.9800	C43'—H43E	0.9800
C20'—H20F	0.9800	C43'—H43F	0.9800
Sn3'—C23'	2.151 (7)	Sn6'—C44'	2.152 (7)
Sn3'—C21'	2.157 (6)	Sn6'—C45'	2.172 (7)
Sn3'—C22'	2.176 (7)	Sn6'—C46'	2.177 (7)
C21'—H21D	0.9800	C44'—H44D	0.9800
C21'—H21E	0.9800	C44'—H44E	0.9800
C21'—H21F	0.9800	C44'—H44F	0.9800
C22'—H22D	0.9800	C45'—H45D	0.9800
C22'—H22E	0.9800	C45'—H45E	0.9800
C22'—H22F	0.9800	C45'—H45F	0.9800
C23'—H23D	0.9800	C46'—H46D	0.9800
C23'—H23E	0.9800	C46'—H46E	0.9800
C23'—H23F	0.9800	C46'—H46F	0.9800
			
C3—Co1—C1	70.3 (2)	C24—Co2—C26	70.8 (2)
C3—Co1—C2	39.6 (2)	C24—Co2—C25	39.0 (2)
C1—Co1—C2	38.5 (2)	C26—Co2—C25	39.4 (2)
C3—Co1—C4	38.4 (2)	C24—Co2—C27	83.0 (2)
C1—Co1—C4	83.1 (2)	C26—Co2—C27	37.9 (2)
C2—Co1—C4	70.8 (2)	C25—Co2—C27	69.9 (2)
C3—Co1—C11	80.9 (2)	C24—Co2—C34	38.24 (18)
C1—Co1—C11	38.1 (2)	C26—Co2—C34	81.00 (19)
C2—Co1—C11	68.7 (2)	C25—Co2—C34	68.77 (19)
C4—Co1—C11	68.30 (19)	C27—Co2—C34	67.99 (18)
C3—Co1—C12	68.4 (2)	C24—Co2—C35	68.27 (19)
C1—Co1—C12	68.8 (2)	C26—Co2—C35	67.7 (2)
C2—Co1—C12	81.8 (2)	C25—Co2—C35	80.68 (19)
C4—Co1—C12	37.86 (19)	C27—Co2—C35	37.62 (17)
C11—Co1—C12	37.15 (17)	C34—Co2—C35	36.63 (16)
C3—Co1—Sn1'	170.6 (2)	C24—Co2—Sn5'	166.4 (3)
C1—Co1—Sn1'	100.5 (2)	C26—Co2—Sn5'	102.3 (3)
C2—Co1—Sn1'	132.9 (2)	C25—Co2—Sn5'	139.3 (3)
C4—Co1—Sn1'	141.2 (2)	C27—Co2—Sn5'	84.6 (3)
C11—Co1—Sn1'	90.71 (18)	C34—Co2—Sn5'	130.5 (3)
C12—Co1—Sn1'	107.26 (19)	C35—Co2—Sn5'	98.4 (3)
C3—Co1—Sn2'	98.8 (2)	C24—Co2—Sn4'	97.6 (3)
C1—Co1—Sn2'	165.9 (2)	C26—Co2—Sn4'	168.4 (3)
C2—Co1—Sn2'	135.2 (2)	C25—Co2—Sn4'	130.4 (3)
C4—Co1—Sn2'	82.8 (2)	C27—Co2—Sn4'	142.9 (3)
C11—Co1—Sn2'	133.4 (2)	C34—Co2—Sn4'	89.6 (3)
C12—Co1—Sn2'	99.2 (2)	C35—Co2—Sn4'	108.3 (3)
Sn1'—Co1—Sn2'	90.02 (19)	Sn5'—Co2—Sn4'	89.0 (3)
C3—Co1—Sn3	86.04 (17)	C24—Co2—Sn6	127.92 (15)
C1—Co1—Sn3	127.76 (16)	C26—Co2—Sn6	85.08 (15)
C2—Co1—Sn3	95.20 (16)	C25—Co2—Sn6	94.87 (14)
C4—Co1—Sn3	106.55 (15)	C27—Co2—Sn6	105.56 (14)
C11—Co1—Sn3	163.89 (13)	C34—Co2—Sn6	163.54 (12)
C12—Co1—Sn3	143.26 (13)	C35—Co2—Sn6	142.22 (12)
C3—Co1—Sn1	144.16 (19)	C24—Co2—Sn4	85.28 (14)
C1—Co1—Sn1	86.74 (15)	C26—Co2—Sn4	141.95 (16)
C2—Co1—Sn1	106.42 (17)	C25—Co2—Sn4	104.72 (15)
C4—Co1—Sn1	166.18 (15)	C27—Co2—Sn4	166.36 (14)
C11—Co1—Sn1	97.96 (12)	C34—Co2—Sn4	98.44 (12)
C12—Co1—Sn1	129.04 (13)	C35—Co2—Sn4	130.42 (12)
Sn3—Co1—Sn1	87.09 (2)	Sn6—Co2—Sn4	87.15 (2)
C3—Co1—Sn2	126.82 (19)	C24—Co2—Sn5	145.11 (15)
C1—Co1—Sn2	146.16 (16)	C26—Co2—Sn5	128.37 (16)
C2—Co1—Sn2	166.12 (17)	C25—Co2—Sn5	167.34 (15)
C4—Co1—Sn2	95.78 (17)	C27—Co2—Sn5	97.77 (15)
C11—Co1—Sn2	110.27 (13)	C34—Co2—Sn5	109.82 (12)
C12—Co1—Sn2	89.63 (14)	C35—Co2—Sn5	91.08 (13)
Sn3—Co1—Sn2	85.14 (2)	Sn6—Co2—Sn5	85.73 (2)
Sn1—Co1—Sn2	87.46 (2)	Sn4—Co2—Sn5	87.95 (2)
C3—Co1—Sn3'	97.4 (2)	C24—Co2—Sn6'	105.7 (4)
C1—Co1—Sn3'	103.7 (2)	C26—Co2—Sn6'	96.5 (3)
C2—Co1—Sn3'	84.7 (2)	C25—Co2—Sn6'	85.8 (4)
C4—Co1—Sn3'	130.8 (2)	C27—Co2—Sn6'	129.0 (3)
C11—Co1—Sn3'	140.25 (19)	C34—Co2—Sn6'	142.7 (3)
C12—Co1—Sn3'	165.27 (19)	C35—Co2—Sn6'	164.1 (3)
Sn1'—Co1—Sn3'	86.31 (18)	Sn5'—Co2—Sn6'	86.5 (3)
Sn2'—Co1—Sn3'	86.25 (19)	Sn4'—Co2—Sn6'	86.8 (3)
C2—C1—C11	122.2 (5)	C25—C24—C34	121.2 (5)
C2—C1—Co1	70.8 (3)	C25—C24—Co2	71.1 (3)
C11—C1—Co1	77.4 (3)	C34—C24—Co2	77.5 (3)
C2—C1—H1	121 (3)	C25—C24—H24	119 (3)
C11—C1—H1	117 (3)	C34—C24—H24	119 (3)
Co1—C1—H1	126 (3)	Co2—C24—H24	131 (3)
C1—C2—C3	118.6 (6)	C24—C25—C26	118.9 (5)
C1—C2—Co1	70.7 (3)	C24—C25—Co2	69.9 (3)
C3—C2—Co1	70.0 (3)	C26—C25—Co2	70.1 (3)
C1—C2—H2	121 (4)	C24—C25—H25	119 (3)
C3—C2—H2	120 (4)	C26—C25—H25	121 (3)
Co1—C2—H2	129 (4)	Co2—C25—H25	128 (3)
C4—C3—C2	121.1 (5)	C27—C26—C25	120.7 (5)
C4—C3—Co1	72.1 (3)	C27—C26—Co2	72.5 (3)
C2—C3—Co1	70.4 (3)	C25—C26—Co2	70.5 (3)
C4—C3—H3	116 (4)	C27—C26—H26	124 (4)
C2—C3—H3	122 (4)	C25—C26—H26	115 (4)
Co1—C3—H3	123 (4)	Co2—C26—H26	134 (4)
C3—C4—C12	120.9 (5)	C26—C27—C35	121.3 (5)
C3—C4—Co1	69.5 (3)	C26—C27—Co2	69.6 (3)
C12—C4—Co1	76.5 (3)	C35—C27—Co2	77.1 (3)
C3—C4—H4	120 (3)	C26—C27—H27	118 (3)
C12—C4—H4	119 (3)	C35—C27—H27	120 (3)
Co1—C4—H4	126 (3)	Co2—C27—H27	127 (3)
C6—C5—C13	120.6 (6)	C29—C28—C36	119.7 (6)
C6—C5—H5	119.7	C29—C28—H28	120.1
C13—C5—H5	119.7	C36—C28—H28	120.1
C5—C6—C7	122.9 (6)	C28—C29—C30	121.8 (6)
C5—C6—H6	118.5	C28—C29—H29	119.1
C7—C6—H6	118.5	C30—C29—H29	119.1
C8—C7—C6	117.7 (5)	C31—C30—C29	120.8 (6)
C8—C7—H7	121.1	C31—C30—H30	119.6
C6—C7—H7	121.1	C29—C30—H30	119.6
C7—C8—C14	122.0 (5)	C30—C31—C37	120.4 (6)
C7—C8—H8	119.0	C30—C31—H31	119.8
C14—C8—H8	119.0	C37—C31—H31	119.8
C14—C9—C11	122.3 (4)	C37—C32—C34	122.3 (4)
C14—C9—H9	118.9	C37—C32—H32	118.8
C11—C9—H9	118.9	C34—C32—H32	118.8
C13—C10—C12	121.6 (5)	C36—C33—C35	122.4 (5)
C13—C10—H10	119.2	C36—C33—H33	118.8
C12—C10—H10	119.2	C35—C33—H33	118.8
C9—C11—C1	123.0 (5)	C32—C34—C35	118.6 (4)
C9—C11—C12	118.8 (5)	C32—C34—C24	122.9 (4)
C1—C11—C12	118.2 (4)	C35—C34—C24	118.5 (4)
C9—C11—Co1	135.6 (3)	C32—C34—Co2	135.6 (3)
C1—C11—Co1	64.4 (3)	C35—C34—Co2	72.7 (3)
C12—C11—Co1	71.5 (3)	C24—C34—Co2	64.2 (3)
C10—C12—C4	123.1 (5)	C33—C35—C34	118.6 (4)
C10—C12—C11	118.7 (5)	C33—C35—C27	123.0 (4)
C4—C12—C11	118.2 (5)	C34—C35—C27	118.4 (5)
C10—C12—Co1	135.5 (4)	C33—C35—Co2	138.5 (4)
C4—C12—Co1	65.7 (3)	C34—C35—Co2	70.6 (3)
C11—C12—Co1	71.4 (3)	C27—C35—Co2	65.3 (3)
C10—C13—C5	121.9 (5)	C33—C36—C28	122.4 (5)
C10—C13—C14	119.9 (5)	C33—C36—C37	119.1 (5)
C5—C13—C14	118.2 (5)	C28—C36—C37	118.5 (5)
C9—C14—C8	122.6 (5)	C32—C37—C31	122.5 (5)
C9—C14—C13	118.8 (5)	C32—C37—C36	119.0 (4)
C8—C14—C13	118.6 (5)	C31—C37—C36	118.5 (5)
C16—Sn1—C17	102.3 (2)	C38—Sn4—C39	99.8 (2)
C16—Sn1—C15	99.3 (2)	C38—Sn4—C40	105.0 (2)
C17—Sn1—C15	104.9 (3)	C39—Sn4—C40	101.9 (2)
C16—Sn1—Co1	112.4 (2)	C38—Sn4—Co2	126.31 (15)
C17—Sn1—Co1	109.18 (16)	C39—Sn4—Co2	112.00 (15)
C15—Sn1—Co1	126.0 (3)	C40—Sn4—Co2	109.04 (16)
Sn1—C15—H15A	109.5	Sn4—C38—H38A	109.5
Sn1—C15—H15B	109.5	Sn4—C38—H38B	109.5
H15A—C15—H15B	109.5	H38A—C38—H38B	109.5
Sn1—C15—H15C	109.5	Sn4—C38—H38C	109.5
H15A—C15—H15C	109.5	H38A—C38—H38C	109.5
H15B—C15—H15C	109.5	H38B—C38—H38C	109.5
Sn1—C16—H16A	109.5	Sn4—C39—H39A	109.5
Sn1—C16—H16B	109.5	Sn4—C39—H39B	109.5
H16A—C16—H16B	109.5	H39A—C39—H39B	109.5
Sn1—C16—H16C	109.5	Sn4—C39—H39C	109.5
H16A—C16—H16C	109.5	H39A—C39—H39C	109.5
H16B—C16—H16C	109.5	H39B—C39—H39C	109.5
Sn1—C17—H17A	109.5	Sn4—C40—H40A	109.5
Sn1—C17—H17B	109.5	Sn4—C40—H40B	109.5
H17A—C17—H17B	109.5	H40A—C40—H40B	109.5
Sn1—C17—H17C	109.5	Sn4—C40—H40C	109.5
H17A—C17—H17C	109.5	H40A—C40—H40C	109.5
H17B—C17—H17C	109.5	H40B—C40—H40C	109.5
C20—Sn2—C18	105.1 (3)	C41—Sn5—C42	99.8 (3)
C20—Sn2—C19	108.1 (3)	C41—Sn5—C43	104.2 (3)
C18—Sn2—C19	98.7 (3)	C42—Sn5—C43	106.4 (2)
C20—Sn2—Co1	106.28 (18)	C41—Sn5—Co2	126.63 (18)
C18—Sn2—Co1	127.0 (2)	C42—Sn5—Co2	110.42 (16)
C19—Sn2—Co1	110.5 (3)	C43—Sn5—Co2	107.81 (16)
Sn2—C18—H18A	109.5	Sn5—C41—H41A	109.5
Sn2—C18—H18B	109.5	Sn5—C41—H41B	109.5
H18A—C18—H18B	109.5	H41A—C41—H41B	109.5
Sn2—C18—H18C	109.5	Sn5—C41—H41C	109.5
H18A—C18—H18C	109.5	H41A—C41—H41C	109.5
H18B—C18—H18C	109.5	H41B—C41—H41C	109.5
Sn2—C19—H19A	109.5	Sn5—C42—H42A	109.5
Sn2—C19—H19B	109.5	Sn5—C42—H42B	109.5
H19A—C19—H19B	109.5	H42A—C42—H42B	109.5
Sn2—C19—H19C	109.5	Sn5—C42—H42C	109.5
H19A—C19—H19C	109.5	H42A—C42—H42C	109.5
H19B—C19—H19C	109.5	H42B—C42—H42C	109.5
Sn2—C20—H20A	109.5	Sn5—C43—H43A	109.5
Sn2—C20—H20B	109.5	Sn5—C43—H43B	109.5
H20A—C20—H20B	109.5	H43A—C43—H43B	109.5
Sn2—C20—H20C	109.5	Sn5—C43—H43C	109.5
H20A—C20—H20C	109.5	H43A—C43—H43C	109.5
H20B—C20—H20C	109.5	H43B—C43—H43C	109.5
C23—Sn3—C21	105.8 (3)	C44—Sn6—C45	99.8 (3)
C23—Sn3—C22	106.6 (3)	C44—Sn6—C46	107.4 (2)
C21—Sn3—C22	100.4 (2)	C45—Sn6—C46	106.1 (2)
C23—Sn3—Co1	109.53 (18)	C44—Sn6—Co2	126.20 (17)
C21—Sn3—Co1	125.91 (17)	C45—Sn6—Co2	107.62 (18)
C22—Sn3—Co1	106.98 (19)	C46—Sn6—Co2	108.02 (17)
Sn3—C21—H21A	109.5	Sn6—C44—H44A	109.5
Sn3—C21—H21B	109.5	Sn6—C44—H44B	109.5
H21A—C21—H21B	109.5	H44A—C44—H44B	109.5
Sn3—C21—H21C	109.5	Sn6—C44—H44C	109.5
H21A—C21—H21C	109.5	H44A—C44—H44C	109.5
H21B—C21—H21C	109.5	H44B—C44—H44C	109.5
Sn3—C22—H22A	109.5	Sn6—C45—H45A	109.5
Sn3—C22—H22B	109.5	Sn6—C45—H45B	109.5
H22A—C22—H22B	109.5	H45A—C45—H45B	109.5
Sn3—C22—H22C	109.5	Sn6—C45—H45C	109.5
H22A—C22—H22C	109.5	H45A—C45—H45C	109.5
H22B—C22—H22C	109.5	H45B—C45—H45C	109.5
Sn3—C23—H23A	109.5	Sn6—C46—H46A	109.5
Sn3—C23—H23B	109.5	Sn6—C46—H46B	109.5
H23A—C23—H23B	109.5	H46A—C46—H46B	109.5
Sn3—C23—H23C	109.5	Sn6—C46—H46C	109.5
H23A—C23—H23C	109.5	H46A—C46—H46C	109.5
H23B—C23—H23C	109.5	H46B—C46—H46C	109.5
C16'—Sn1'—C17'	102.3 (5)	C38'—Sn4'—C39'	99.8 (4)
C16'—Sn1'—C15'	99.3 (4)	C38'—Sn4'—C40'	105.0 (5)
C17'—Sn1'—C15'	104.9 (5)	C39'—Sn4'—C40'	101.9 (5)
C16'—Sn1'—Co1	113 (4)	C38'—Sn4'—Co2	120 (4)
C17'—Sn1'—Co1	113 (2)	C39'—Sn4'—Co2	122 (4)
C15'—Sn1'—Co1	122 (3)	C40'—Sn4'—Co2	106 (4)
Sn1'—C15'—H15D	109.5	Sn4'—C38'—H38D	109.5
Sn1'—C15'—H15E	109.5	Sn4'—C38'—H38E	109.5
H15D—C15'—H15E	109.5	H38D—C38'—H38E	109.5
Sn1'—C15'—H15F	109.5	Sn4'—C38'—H38F	109.5
H15D—C15'—H15F	109.5	H38D—C38'—H38F	109.5
H15E—C15'—H15F	109.5	H38E—C38'—H38F	109.5
Sn1'—C16'—H16D	109.5	Sn4'—C39'—H39D	109.5
Sn1'—C16'—H16E	109.5	Sn4'—C39'—H39E	109.5
H16D—C16'—H16E	109.5	H39D—C39'—H39E	109.5
Sn1'—C16'—H16F	109.5	Sn4'—C39'—H39F	109.5
H16D—C16'—H16F	109.5	H39D—C39'—H39F	109.5
H16E—C16'—H16F	109.5	H39E—C39'—H39F	109.5
Sn1'—C17'—H17D	109.5	Sn4'—C40'—H40D	109.5
Sn1'—C17'—H17E	109.5	Sn4'—C40'—H40E	109.5
H17D—C17'—H17E	109.5	H40D—C40'—H40E	109.5
Sn1'—C17'—H17F	109.5	Sn4'—C40'—H40F	109.5
H17D—C17'—H17F	109.5	H40D—C40'—H40F	109.5
H17E—C17'—H17F	109.5	H40E—C40'—H40F	109.5
C20'—Sn2'—C18'	105.1 (5)	C41'—Sn5'—C42'	99.8 (5)
C20'—Sn2'—C19'	108.1 (5)	C41'—Sn5'—C43'	104.2 (5)
C18'—Sn2'—C19'	98.7 (5)	C42'—Sn5'—C43'	106.4 (5)
C20'—Sn2'—Co1	116 (2)	C41'—Sn5'—Co2	125 (5)
C18'—Sn2'—Co1	122 (3)	C42'—Sn5'—Co2	107 (4)
C19'—Sn2'—Co1	105 (3)	C43'—Sn5'—Co2	112 (4)
Sn2'—C18'—H18D	109.5	Sn5'—C41'—H41D	109.5
Sn2'—C18'—H18E	109.5	Sn5'—C41'—H41E	109.5
H18D—C18'—H18E	109.5	H41D—C41'—H41E	109.5
Sn2'—C18'—H18F	109.5	Sn5'—C41'—H41F	109.5
H18D—C18'—H18F	109.5	H41D—C41'—H41F	109.5
H18E—C18'—H18F	109.5	H41E—C41'—H41F	109.5
Sn2'—C19'—H19D	109.5	Sn5'—C42'—H42D	109.5
Sn2'—C19'—H19E	109.5	Sn5'—C42'—H42E	109.5
H19D—C19'—H19E	109.5	H42D—C42'—H42E	109.5
Sn2'—C19'—H19F	109.5	Sn5'—C42'—H42F	109.5
H19D—C19'—H19F	109.5	H42D—C42'—H42F	109.5
H19E—C19'—H19F	109.5	H42E—C42'—H42F	109.5
Sn2'—C20'—H20D	109.5	Sn5'—C43'—H43D	109.5
Sn2'—C20'—H20E	109.5	Sn5'—C43'—H43E	109.5
H20D—C20'—H20E	109.5	H43D—C43'—H43E	109.5
Sn2'—C20'—H20F	109.5	Sn5'—C43'—H43F	109.5
H20D—C20'—H20F	109.5	H43D—C43'—H43F	109.5
H20E—C20'—H20F	109.5	H43E—C43'—H43F	109.5
C23'—Sn3'—C21'	105.8 (5)	C44'—Sn6'—C45'	99.8 (5)
C23'—Sn3'—C22'	106.6 (5)	C44'—Sn6'—C46'	107.4 (5)
C21'—Sn3'—C22'	100.4 (4)	C45'—Sn6'—C46'	106.1 (5)
C23'—Sn3'—Co1	108 (2)	C44'—Sn6'—Co2	126 (4)
C21'—Sn3'—Co1	133 (2)	C45'—Sn6'—Co2	110 (4)
C22'—Sn3'—Co1	99 (2)	C46'—Sn6'—Co2	106 (4)
Sn3'—C21'—H21D	109.5	Sn6'—C44'—H44D	109.5
Sn3'—C21'—H21E	109.5	Sn6'—C44'—H44E	109.5
H21D—C21'—H21E	109.5	H44D—C44'—H44E	109.5
Sn3'—C21'—H21F	109.5	Sn6'—C44'—H44F	109.5
H21D—C21'—H21F	109.5	H44D—C44'—H44F	109.5
H21E—C21'—H21F	109.5	H44E—C44'—H44F	109.5
Sn3'—C22'—H22D	109.5	Sn6'—C45'—H45D	109.5
Sn3'—C22'—H22E	109.5	Sn6'—C45'—H45E	109.5
H22D—C22'—H22E	109.5	H45D—C45'—H45E	109.5
Sn3'—C22'—H22F	109.5	Sn6'—C45'—H45F	109.5
H22D—C22'—H22F	109.5	H45D—C45'—H45F	109.5
H22E—C22'—H22F	109.5	H45E—C45'—H45F	109.5
Sn3'—C23'—H23D	109.5	Sn6'—C46'—H46D	109.5
Sn3'—C23'—H23E	109.5	Sn6'—C46'—H46E	109.5
H23D—C23'—H23E	109.5	H46D—C46'—H46E	109.5
Sn3'—C23'—H23F	109.5	Sn6'—C46'—H46F	109.5
H23D—C23'—H23F	109.5	H46D—C46'—H46F	109.5
H23E—C23'—H23F	109.5	H46E—C46'—H46F	109.5
			
C11—C1—C2—C3	7.7 (7)	C34—C24—C25—C26	9.7 (7)
Co1—C1—C2—C3	−52.9 (4)	Co2—C24—C25—C26	−52.0 (4)
C11—C1—C2—Co1	60.6 (4)	C34—C24—C25—Co2	61.7 (4)
C1—C2—C3—C4	−0.2 (7)	C24—C25—C26—C27	−2.5 (7)
Co1—C2—C3—C4	−53.4 (4)	Co2—C25—C26—C27	−54.4 (4)
C1—C2—C3—Co1	53.2 (4)	C24—C25—C26—Co2	51.8 (4)
C2—C3—C4—C12	−6.4 (8)	C25—C26—C27—C35	−6.2 (7)
Co1—C3—C4—C12	−59.0 (4)	Co2—C26—C27—C35	−59.6 (4)
C2—C3—C4—Co1	52.6 (4)	C25—C26—C27—Co2	53.5 (4)
C13—C5—C6—C7	−0.8 (11)	C36—C28—C29—C30	0.0 (10)
C5—C6—C7—C8	0.5 (10)	C28—C29—C30—C31	−2.0 (10)
C6—C7—C8—C14	0.4 (8)	C29—C30—C31—C37	0.5 (9)
C14—C9—C11—C1	177.3 (4)	C37—C32—C34—C35	2.0 (7)
C14—C9—C11—C12	−1.0 (7)	C37—C32—C34—C24	−178.3 (4)
C14—C9—C11—Co1	91.4 (6)	C37—C32—C34—Co2	96.2 (6)
C2—C1—C11—C9	173.3 (4)	C25—C24—C34—C32	172.2 (4)
Co1—C1—C11—C9	−129.3 (4)	Co2—C24—C34—C32	−129.2 (4)
C2—C1—C11—C12	−8.4 (7)	C25—C24—C34—C35	−8.1 (7)
Co1—C1—C11—C12	49.0 (4)	Co2—C24—C34—C35	50.4 (4)
C2—C1—C11—Co1	−57.4 (4)	C25—C24—C34—Co2	−58.6 (4)
C13—C10—C12—C4	−179.4 (5)	C36—C33—C35—C34	0.6 (8)
C13—C10—C12—C11	0.6 (8)	C36—C33—C35—C27	178.9 (5)
C13—C10—C12—Co1	−91.6 (7)	C36—C33—C35—Co2	−92.0 (6)
C3—C4—C12—C10	−174.6 (5)	C32—C34—C35—C33	−2.4 (7)
Co1—C4—C12—C10	129.7 (5)	C24—C34—C35—C33	177.9 (4)
C3—C4—C12—C11	5.5 (7)	Co2—C34—C35—C33	−135.4 (4)
Co1—C4—C12—C11	−50.2 (4)	C32—C34—C35—C27	179.1 (4)
C3—C4—C12—Co1	55.6 (4)	C24—C34—C35—C27	−0.5 (6)
C9—C11—C12—C10	0.2 (7)	Co2—C34—C35—C27	46.1 (4)
C1—C11—C12—C10	−178.2 (4)	C32—C34—C35—Co2	133.0 (4)
Co1—C11—C12—C10	−132.3 (5)	C24—C34—C35—Co2	−46.7 (4)
C9—C11—C12—C4	−179.9 (4)	C26—C27—C35—C33	−170.7 (5)
C1—C11—C12—C4	1.7 (6)	Co2—C27—C35—C33	133.2 (5)
Co1—C11—C12—C4	47.6 (4)	C26—C27—C35—C34	7.6 (7)
C9—C11—C12—Co1	132.5 (4)	Co2—C27—C35—C34	−48.5 (4)
C1—C11—C12—Co1	−45.9 (4)	C26—C27—C35—Co2	56.1 (4)
C12—C10—C13—C5	178.5 (5)	C35—C33—C36—C28	−174.8 (5)
C12—C10—C13—C14	−0.5 (8)	C35—C33—C36—C37	1.7 (8)
C6—C5—C13—C10	−178.9 (6)	C29—C28—C36—C33	179.8 (6)
C6—C5—C13—C14	0.2 (9)	C29—C28—C36—C37	3.3 (8)
C11—C9—C14—C8	−179.0 (4)	C34—C32—C37—C31	179.4 (5)
C11—C9—C14—C13	1.0 (7)	C34—C32—C37—C36	0.2 (7)
C7—C8—C14—C9	179.1 (5)	C30—C31—C37—C32	−176.3 (5)
C7—C8—C14—C13	−1.0 (7)	C30—C31—C37—C36	2.9 (8)
C10—C13—C14—C9	−0.3 (7)	C33—C36—C37—C32	−2.1 (7)
C5—C13—C14—C9	−179.4 (5)	C28—C36—C37—C32	174.5 (5)
C10—C13—C14—C8	179.8 (5)	C33—C36—C37—C31	178.7 (5)
C5—C13—C14—C8	0.7 (7)	C28—C36—C37—C31	−4.7 (7)
